# Interpretable patent recommendation with knowledge graph and deep learning

**DOI:** 10.1038/s41598-023-28766-y

**Published:** 2023-02-14

**Authors:** Han Chen, Weiwei Deng

**Affiliations:** 1 College of Teacher Education, South China Normal University, Guangzhou, China; 2 School of Economics and Management, South China Normal University, Guangzhou, China

**Keywords:** Information technology, Scientific data

## Abstract

Patent transfer is a common practice for companies to obtain competitive advantages. However, they encounter the difficulty of selecting suitable patents because the number of patents is increasingly large. Many patent recommendation methods have been proposed to ease the difficulty, but they ignore patent quality and cannot explain why certain patents are recommended. Patent quality and recommendation explanations affect companies’ decision-making in the patent transfer context. Failing to consider them in the recommendation process leads to less effective recommendation results. To fill these gaps, this paper proposes an interpretable patent recommendation method based on knowledge graph and deep learning. The proposed method organizes heterogeneous patent information as a knowledge graph. Then it extracts connectivity and quality features from the knowledge graph for pairs of patents and companies. The former features indicate the relevance of the pairs while the latter features reflect the quality of the patents. Based on the features, we design an interpretable recommendation model by combining a deep neural network with a relevance propagation technique. We conduct experiments with real-world data to evaluate the proposed method. Recommendation lists with varying lengths show that the average precision, recall, and mean average precision of the proposed method are 0.596, 0.636, and 0.584, which improve corresponding performance of best baselines by 7.28%, 18.35%, and 8.60%, respectively. Besides, our method interprets recommendation results by identifying important features leading to the results.

## Introduction

Patent transfer refers to the movement of patent rights from one party to another. With the advent of open innovation, patent transfer has become a common practice for companies to obtain competitive advantages. Both patent owners and demanders have incentives to transfer patents. For the former, patent transfer provides monetary benefits (e.g., royalty payment) and strategic benefits (e.g., strengthening companies’ product market and technological positions)^1^. For the latter, patent transfer reduces the risks and costs associated with technological innovations, provides industry standards and advanced technologies, and facilitates technological learning^2^. Previous research has shown that patent transfer has significant impacts not only on companies’ performance but also on national economies^2,3^. For example, Han and Lee^4^ demonstrated that patent transfer significantly increases the market value of firms in Korea. Kwon^5^ found that firms’ patent transfer makes their rivals deter innovative activities. Roessner et al.^6^ combined university licensing data of the United States (US) from 1996 to 2010 with economic input–output models and found that the contribution of university licensing to the US economy during that period was at least 162.1 billion dollars.

Due to the importance of patent transfer, online patent platforms have been developed to facilitate the transfer process^7,8^. However, patent demanders still face an information overload problem as the number of patents is getting large. The number of yearly granted patents has exceeded 0.5 million since 2005 and reached 1.5 million in 2019 as recorded by the World Intellectual Property Organization (https://www.wipo.int/publications/en/details.jsp?id=4526&plang = EN). Consequently, the cost of searching for suitable patents is high. Many patent recommendation methods have been developed to suggest suitable patents to users^9–13^. However, previous methods fail to consider patent quality and explain recommendation results. The quality of patents reflects their value and is essential to firms. Previous studies show that patent quality is positively correlated to firms’ stock returns^14^, high-quality patents can protect firms from patent trolls and product infringement^15^, and the uncertainty of patent quality may cost enterprises billions^16^. Interpretability of the recommendation results also matters. Patents have complex information and cost companies much to select a suitable one. The interpretability increases information transparency and thus promotes persuasiveness and acceptance of recommender systems^17^. Besides, interpretable recommendations benefit users and platforms because of the improved user-friendliness^18^. Therefore, providing interpretable recommendations is necessary to improve companies’ decision-making in selecting suitable patents.

To bridge these gaps, this research proposes an interpretable patent recommendation method to facilitate companies to select suitable patents. The proposed method organizes heterogeneous patent information as a knowledge graph, a graph-structured knowledge base that enables efficient integration and semantic interpretation of heterogeneous information^19^. It then extracts two types of features from the knowledge graph, i.e., connectivity features and quality features that indicate the relevance of company-patent pairs and patent quality. Last, the proposed method builds an interpretable deep learning model for recommending patents and providing explanations to companies. Deep learning is popular in personalized recommendations for its superior performance, but it lacks interpretability. Therefore, this research develops an interpretable deep learning model by combining a deep neural network (DNN) with a layer-wise relevance propagation technique (LRP)^20^. DNN predicts the probabilities that target companies will select candidate patents while LRP generates interpretations by identifying the features that contribute the most to the predictions. The proposed method is evaluated with data obtained from the United States Patent and Trademark Office (USPTO, https://www.uspto.gov/learning-and-resources/electronic-data-products/patent-assignment-dataset) and PatentsView (http://www.patentsview.org/download/) databases. The evaluation results show that the proposed method not only outperforms the state-of-the-art patent recommendation methods in terms of the precision, recall, and mean average precision (MAP) measures but also provides valid interpretations.

The rest of this paper is organized as follows. Section "Related work" reviews studies on patent recommendations and patent quality analysis. Section "The proposed patent recommendation method" presents the proposed knowledge graph-based method for interpretable patent recommendations. Section "Experimental evaluation" introduces the details of the experimental evaluation. Section "Results and discussions" presents and discusses the evaluation results. Section "Conclusions" concludes this research with major contributions and possible future works.

## Related work

### Patent recommendations

Recommendation methods have been widely used to solve information overload problems in different contexts by proactively delivering personalized recommendations^21^. In the patent domain, recommendation methods have also been proposed to help users to find suitable patents. Previous patent recommendation methods differ mainly in application contexts, the information involved in the recommendations, and the techniques applied for the recommendations. The application contexts mainly contain query-driven patent search and patent transfer contexts^8^. The former provides specific user needs like keywords or patent documents while the latter requires the mining of company needs^9,22^. Patents have heterogeneous information that can be used for recommendation, including texts (e.g., patent titles and abstracts), categories (e.g., the International Patent Classification), interactions (e.g., patent searching and assignment behavior of users), citations, and inventors of patents^23^. Besides, the applied techniques of patent recommendations can be divided into content-based filtering (CB), collaborative filtering (CF), and hybrid methods^12,24^. Table 1 summarizes major patent recommendation studies based on the above aspects.

**Table 1:** Major studies on patent recommendations.

Studies	Contexts	Involved information						Applied techniques	Interpretability
		Texts	Categories	Citations	Inventors	Interactions	Quality		
Chen and Chiu ^25^	C1		√					CB	
Ji et al.^26^	C1	√	√			√		Hybrid	
Sooyoung Oh et al.^27^	C1	√	√	√	√			Hybrid	
Trappey et al.^28^	C1					√		CF	
Krestel and Smyth^29^	C1	√						CB	
Oh et al.^30^	C1			√				CF	
Mahdabi and Crestani^31^	C1	√	√	√	√			Hybrid	
Fu et al.^23^	C1	√	√	√	√	√		Hybrid	
Deng et al.^32^	C1	√						CB	
Rui and Min^33^	C1	√						CB	
Chen et al.^9^	C1	√			√			CB	
Trappey et al.^12^	C1	√						CB	
Wang et al.^8^	C2	√	√	√	√	√		Hybrid	
He et al.^10^	C2		√			√		Hybrid	
Deng and Ma (2021)	C2	√						CB	
Du et al.^22^	C2	√	√	√	√	√		Hybrid	
The proposed method	C2	√	√	√	√	√	√	Hybrid	√

C1 is the query-driven patent search context, C2 is the patent transfer context, CB represents content-based recommendation methods, and CF represents collaborative filtering recommendation methods.

Several conclusions and research gaps can be identified from Table 1. First, previous studies mainly focus on the query-driven patent search context and pay less attention to the patent transfer context. In the former context, users provide explicit needs and care little about patent quality. For example, patent inventors provide key terms to search relevant prior arts before applying patents^28^. Patent examiners use patent applications to search missing references and examine the novelty of the applications^23^. Patent users identify similar patents for a given target patent^9^. Only several methods have been proposed for the patent transfer context and most of them were proposed recently. This situation indicates the increasing importance of patent transfer and calls for greater attention to improving patent transfer.

Second, previous studies have used various patent information but ignored patent quality. As shown in Table 1, previous studies on patent recommendations have different types of patent information, including texts, categories, citations, inventors, and interactions. However, they failed to consider patent quality, which is important to companies’ value and competitiveness^14,15^. Ignoring the quality of acquired patents can impede business and result in costly lawsuits^16^. Therefore, incorporating patent quality is imperative to recommending suitable patents to companies.

Third, CB and hybrid methods gain more popularity than CF methods in patent recommendation. CF methods recommend patents based on like-minded users. For example, Trappey et al.^28^ used a user-based CF method to identify users with similar patent searching behaviors and recommend patents that are liked by similar users. CF methods are less suitable in the technology transfer context because most patents are transferred only once to only one company. Consequently, CF methods suffer from the data sparsity problem in the technology transfer context. CB methods recommend patents that are similar to the patents liked by target users. For example, Krestel and Smyth^29^ used a latent Dirichlet allocation model to recommend patents with topics similar to a query patent. Chen and Chiu^25^ developed an IPC-based vector space model to find similar patents for a given patent. CB methods are suitable for the query-driven context because it mainly requires the matching between patents and queries. However, CB methods have difficulty in involving heterogeneous information and face an over-specialization problem, i.e., they recommend only patents with features previously liked by target users. Hybrid methods combine different methods to overcome their disadvantages. One frequently used hybrid mechanism is to learn a unified model based on the collaborative and content information. For example, Mahdabi and Crestani^31^ developed a citation query model which combines information including patent text, citations, categories, and inventors. Hybrid methods are popular in patent recommendation because of their advantages in overcoming the drawbacks of CF and CB methods. Consequently, this study proposes a hybrid method that incorporates heterogeneous patent information for patent recommendation.

Last, previous studies ignore the interpretability of patent recommendations. Given the importance of interpretability to companies’ decision-making^17,18^, there is a need for endowing recommendation results with interpretability. Therefore, this study develops an interpretable patent recommendation method, which can identify the features that contribute the most to each recommendation. Based on the identified features, we can explain why a specific patent is recommended to a specific company.

### Patent quality analysis

Patent quality analysis focuses on identifying potential indicators of patent quality. Multiple indicators of patent quality have been proposed^34^. Among the proposed indicators, we summarize eight frequently used indicators, i.e., the number of forward citations, the number of backward citations, the number of patent claims, patent scope, previous transfer, patent family size, patent generality, and patent originality.

Forward citations are the citations that patents receive. A higher number of forward citations indicates a greater value of current inventions and their importance for subsequent technologies^35,36^. A patent’s backward citations are the references the patent has. More references indicate the more developed a technological field is and more incrementally the patent contributes to the field. Previous studies^36,37^ have consistently shown that there is a positive correlation between the number of backward citations and patent quality. A patent’s claims delineate the property rights protected by the patent and a larger number of patent claims indicates higher patent quality^38^. Patent scope is defined as the number of distinct patent classes to which patents are allocated. The broader the patent scope, the more attractive the patents’ exclusive rights, and hence the higher quality of the patents^38^. Previous transfer refers to the number of times a patent has been previously traded. It indicates the patent’s value as recognized by the technological market^39^. Patent family size is the number of countries in which a patented invention is protected. A larger patent family size indicates a higher quality of patents because the patented technologies are protected in a broader geographical scope^40^. Patent generality measures the generality of a patent’s impact. A patent has a general impact if it is cited by other patents that belong to a wide range of technological fields^40,41^. Patent originality measures the originality of a patent. A patent is believed to be more original if it relies on diverse knowledge sources (i.e., its backward citations belong to a wide range of technological fields)^40,41^. According to previous research, this study uses these eight indicators to measure patent quality.

## The proposed patent recommendation method

### Overview of the proposed method

**Figure 1:**
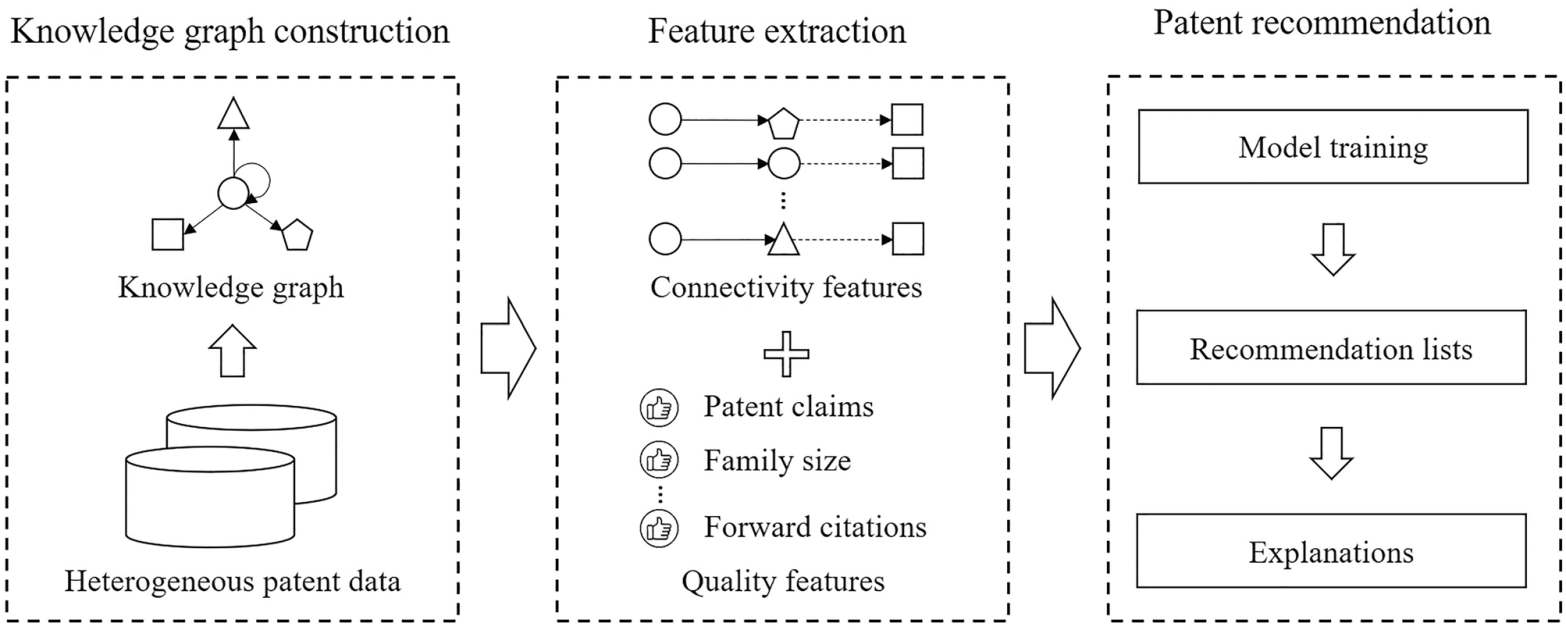
Overview of the proposed patent recommendation method.

Figure 1 shows the framework of the proposed patent recommendation method. In the proposed method, we organize heterogeneous patent information as a patent knowledge graph that stores the information in the form of semantic relations between entities. The proposed method comprises three modules, namely, knowledge graph construction, feature extraction, and patent recommendation. The first module extracts patent and company information to construct a knowledge graph. The second module extracts, from the knowledge graph, connectivity and quality features that indicate the relevance between companies and patents and the quality of patents, respectively. Given the extracted features, the third module develops an interpretable patent recommendation model to recommend suitable patents and to provide interpretations to target companies. The following subsections present the details of these three modules.

### Knowledge graph construction

#### Concepts and definitions

Definition 1 (Knowledge Graph). A knowledge graph is a graph-structured knowledge base denoted as $\mathcal{G}=\left\{\left(h,r,t\right)\right\}\subseteq \mathcal{E}\times \mathcal{R}\times \mathcal{E}$, where $\mathcal{E}$ is a set of entities, $\mathcal{R}$ is a set of relations, $h,t\in \mathcal{E}$, and $r\in \mathcal{R}$. A triple $\left(h,r,t\right)$ represents the fact that there is a relation $r$ between entities $h$ and $t$. For example, $\left(paten{t}_{i},has term,ter{m}_{j}\right)$ indicates the fact that $paten{t}_{i}$ contains $ter{m}_{j}$.

Definition 2 (Meta Path). A meta path is a type of composite relations between two types of entities. It is denoted as $P = \left( {H,r_{1} ,E_{1} ,r_{2} , \ldots ,r_{l} ,T} \right)$, where $H$ and $T$ are the types of head and tail entities respectively, ${r}_{l}\in \mathcal{R}$ is a relation, ${E}_{1}$ is the type of entities reached through ${r}_{1}$, and $l$ denotes the length of the meta path. Meta paths capture the semantic connections between entities and can be used to measure relevance between entities. For example, (*Patent*, *has_term*, *Term*, *has_term*, *Patent*, *is_transfered_to*, *Company*) is a meta path between patents and companies. The meta path indicates that the head patents are relevant to the companies because the head patents have the same terms as the patents transferred to the companies. A meta path can contain multiple concrete paths. For example, (*patent*_1_, *has_term*, *term*_1_, *has_term*, *patent*_2_, *is_transfered_to*, *company*_1_) and (*patent*_1_, *has_term*, *term*_2_, *has_term*, *patent*_2_, *is_transfered_to*, *company*_1_) are two concrete paths that belong to the above meta path.

#### Knowledge graph

**Figure 2:**
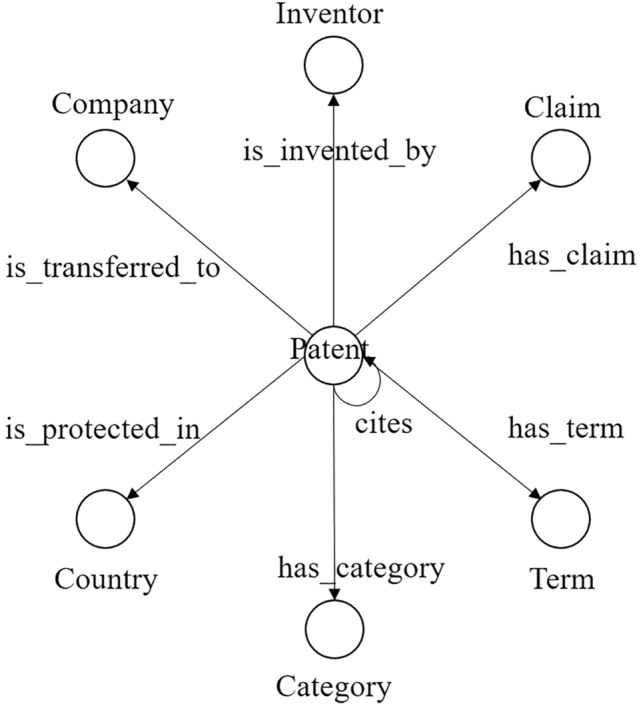
The graph schema of the knowledge graph.

A domain-specific knowledge graph is constructed for patent recommendation in the current context. The types of facts in the knowledge graph are shown in Fig. 2. Specifically, the knowledge graph comprises seven types of entities (i.e., patents, companies, inventors, terms, categories, countries, and claims) and eight types of relations (i.e., ${patent}_{i}$ cites ${patent}_{j}$, ${patent}_{i}$ is invented by ${inventor}_{j}$, ${patent}_{i}$ is transferred to ${company}_{j}$, ${patent}_{i}$ has term ${term}_{j}$, ${patent}_{i}$ has category ${category}_{j}$, ${patent}_{i}$ is protected in ${country}_{j}$, and ${patent}_{i}$ has claim ${claim}_{j}$).

The knowledge graph is constructed by extracting the above types of entities and relations from patent databases. For example, we can extract, from Google’s patent database, two patents “5,208,847” and “4,893,327”, a company “Rockstar Bidco”, an inventor “Daniel L. Allen”, two terms “cellular” and “network”, two categories “H04W16/00” and “H04W8/26”, a country “the US”, and a claim “A method for providing an overlay cellular network”. When extracting terms from patents, text-processing techniques developed by Stanford^42^ are used to converse cases, remove stop words, standardize derivate words, etc. We can further extract relations between these entities, i.e., “5,208,847” cites “4,893,327”, “5,208,847” is transferred to “Rockstar Bidco”, “5,208,847” is invented by “Daniel L. Allen”, “5,208,847” has terms “cellular” and “network”, “5,208,847” has categories “H04W16/00” and “H04W8/26”, “5,208,847” is protected in “the US”, and “5,208,847” has a claim “A method for providing an overlay cellular network”. The knowledge graph is an accumulative knowledge base and can include more facts to be extracted.

Patent data except terms are structured and can be imported from public databases (e.g., the USPTO and PatentsView databases) to the knowledge graph. For patent terms, this study uses the rapid automatic keyword extraction (RAKE) method^43^ to extract three representative terms from the titles and abstracts of patents. RAKE is an unsupervised and domain-independent method for term extraction. It has higher accuracy and efficiency than other term extraction methods and thus is selected in this study.

### Feature extraction

#### Connectivity features

Connectivity features are the connections between patents and companies and indicate their relevance based on various connecting entities. For example, a path (*patent*_1_, *cites*, *patent*_2_, *is_transfered_to*, *company*_1_) indicates that *patent*_1_ is relevant to *company*_1_ because it cites a patent transferred to the company. We define meta paths that connect companies to patents as the connectivity features. Meta paths involving claims are not considered because patents have unique claims, meaning that these meta paths have no concrete paths connecting any patent to any company. Besides, this study considers only meta paths within a certain length (e.g., 3) because long meta paths provide trivial information while causing high computational costs^44^. Therefore, from the constructed knowledge graph, this study extracts six connectivity features, which are presented along with their descriptions in Table 2.

**Table 2:** Connectivity features and their descriptions.

No	Connectivity features	Descriptions
*P*_1_	$$Patent\stackrel{has term}{\to }Term\stackrel{has term}{\leftarrow }Patent\stackrel{is transferred to}{\to }Company$$	The head patents have the same terms as the patents that were transferred to the companies
*P*_2_	$$Patent\stackrel{has category}{\to }Category\stackrel{has category}{\leftarrow }Patent\stackrel{is transferred to}{\to }Company$$	The head patents have the same categories as the patents that were transferred to the companies
*P*_3_	$$Patent\stackrel{cites}{\leftrightarrow }Patent\stackrel{is transferred to}{\to }Company$$	The head patents have citation relation with the patents that were transferred to the companies
*P*_4_	$$Patent\stackrel{cites}{\leftrightarrow }Patent\stackrel{cites}{\leftrightarrow }Patent\stackrel{is transferred to}{\to }Company$$	The head patents have indirect citation relation with the patents that were transferred to the companies
*P*_5_	$$Patent\stackrel{is invented by}{\to }Inventor\stackrel{is invented by}{\leftarrow }Patent\stackrel{is transferred to}{\to }Company$$	The head patents share the same inventors as the patents that were transferred to the companies
*P*_6_	$$Patent\stackrel{is transferred to}{\to }Company\stackrel{is transferred to}{\leftarrow }Patent\stackrel{is transferred to}{\to }Company$$	The head patents share the same assignees with the patents that were transferred to the tail companies
*P*_7_	$$Patent\stackrel{is protected in}{\to }Country\stackrel{is protected in}{\leftarrow }Patent\stackrel{is transferred to}{\to }Company$$	The head patents are protected in the same countries as the patents that were transferred to the tail companies

The connectivity features need to be quantified before being used for patent recommendations. Given a patent-company pair $\left({patent}_{i},{company}_{j}\right)$, let $\#{path}_{ij}\left({P}_{k}\right)$ be the number of concrete paths that connect ${patent}_{i}$ to ${company}_{j}$ and belong to connectivity feature ${P}_{k}$. Then, this study quantifies the feature between ${patent}_{i}$ and ${company}_{j}$ as follows:

1 $${x}_{ij}\left({P}_{k}\right)=\frac{\#{path}_{ij}\left({P}_{k}\right)-{min}_{r\in {Pat}_{j}}\left(\#{path}_{rj}\left({P}_{k}\right)\right)}{{max}_{r\in Pat}\left(\#{path}_{rj}\left({P}_{k}\right)\right)-{min}_{r\in Pat}\left(\#{path}_{rj}\left({P}_{k}\right)\right)}$$

where ${Pat}_{j}$ is a set of candidate patents for ${company}_{j}$, ${max}_{r\in {Pat}_{j}}\left(\#{path}_{rj}\left({P}_{k}\right)\right)$ and ${min}_{r\in {Pat}_{j}}\left(\#{path}_{rj}\left({P}_{k}\right)\right)$ are the maximum and minimum numbers of concrete paths that belong to ${P}_{k}$ and connect any ${patent}_{r}$ in $Pat$ to ${company}_{j}$, and ${x}_{ij}\left({P}_{k}\right)$ equals 0 if ${max}_{r\in Pat}\left(\#{path}_{rj}\left({P}_{k}\right)\right)={min}_{r\in {Pat}_{j}}\left(\#{path}_{rj}\left({P}_{k}\right)\right)$. ${x}_{ij}\left({P}_{k}\right)$ reflects the connection strength between ${patent}_{i}$ and ${company}_{j}$ through ${P}_{k}$ considering all candidate patents that can connect ${company}_{j}$ via ${P}_{k}$.

#### Quality features

The quality features indicate the quality of patents. As discussed in Section "Patent quality analysis", eight quality indicators are used to quantify patent quality and are mathematically defined as follows.

First, the number of forward citations (NFC). It suffers from the anti-recency problem that older patents have longer periods to accumulate forward citations than newer patents. To address the problem, we consider the publication time of patents and define NFC as follows:

2 $$ NFC_{{patent_{i} }} = \frac{{\mathop \sum \nolimits_{j = 1}^{{\left| {Patent\_set} \right|}} \# \left( {patent_{j} ,cites,patent_{i} } \right)}}{y}, $$

where $Patent\_set$ is the set of patents in the knowledge graph, $\#\left({patent}_{j},cites,{patent}_{i}\right)$ equals 1 if $\left({patent}_{j},cites,{patent}_{i}\right)$ exists in the knowledge graph and 0 otherwise, ${\sum }_{j=1}^{\left|Patent\right|}\#\left({patent}_{j},cites,{patent}_{i}\right)$ counts the number of patents that cite ${patent}_{i}$, and $y$ is the number of years since the publication of ${patent}_{i}$.

Second, the number of backward citations (NBC). It is defined as follows:

3 $$ NBC_{{patent_{i} }} = \mathop \sum \limits_{j = 1}^{{\left| {Patent\_set} \right|}} \# \left( {patent_{i} ,cites,patent_{j} } \right), $$

where ${\sum }_{j=1}^{\left|Patent\_set\right|}\#\left({patent}_{i},cites,{patent}_{j}\right)$ counts the number of patents cited by ${patent}_{i}$.

Third, the number of claims (NCL). It is defined as follows:

4 $$ NCL_{{patent_{i} }} = \mathop \sum \limits_{j = 1}^{{\left| {Claim\_set} \right|}} \# \left( {patent_{i} ,has\_claim,claim_{j} } \right), $$

where $Claim\_set$ is the set of claims in the knowledge graph and ${\sum }_{j=1}^{\left|Claim\_set\right|}\#\left({patent}_{i},has\_claim,{claim}_{j}\right)$ counts the number of claims contained by ${patent}_{i}$.

Fourth, patent scope (PS). It is defined as follows:

5 $${PS}_{{patent}_{i}}={\sum }_{j=1}^{\left|Category\_set\right|}\#\left({patent}_{i},has\_category,{category}_{j}\right),$$

where $Category\_set$ is the set of categories in the knowledge graph and ${\sum }_{j=1}^{\left|Category\_set\right|}\#\left({patent}_{i},has\_category,{category}_{j}\right)$ counts the number of categories owned by ${patent}_{i}$.

Fifth, previous transfers (PT). It is defined as follows:

6 $$ PT_{{patent_{i} }} = \mathop \sum \limits_{j = 1}^{{\left| {Company\_set} \right|}} \# \left( {patent_{i} ,is\_transferred\_to,company_{j} } \right), $$

where $Company\_set$ is the set of companies in the knowledge graph and ${\sum }_{j=1}^{\left|Company\_set\right|}\#\left({patent}_{i},is\_transferred\_to,{company}_{j}\right)$ counts the number of companies to which ${patent}_{i}$ has been transferred.

Sixth, patent family size (PFS). It is defined as follows:

7 $${PFS}_{{patent}_{i}}={\sum }_{j=1}^{\left|Country\_set\right|}\#\left({patent}_{i},is\_protected\_in,{country}_{j}\right),$$

where $Country\_set$ is the set of countries in the knowledge graph and ${\sum }_{j=1}^{\left|Country\_set\right|}\#\left({patent}_{i},is\_protected\_in,{country}_{j}\right)$ counts the number of companies in which ${patent}_{i}$ is protected.

Seventh, patent generality (PG). Eighth, patent originality (PO). These two indicators are defined as follows:

8 $$ PG_{{patent_{i} }} = \frac{{\left| {\left\{ {category{|}\# \left( {patent_{m} ,has_{category} ,category} \right) = 1,patent_{m} \in Patent\_set_{i,f} } \right\}} \right|}}{{\left| {Patent\_set_{i,f} } \right|}}, $$

9 $$ PO_{{patent_{i} }} = \frac{{\left| {\left\{ {category{|}\# \left( {patent_{m} ,has_{category} ,category} \right) = 1,patent_{m} \in Patent\_set_{i,b} } \right\}} \right|}}{{\left| {Patent\_set_{i,b} } \right|}}, $$

where ${Patent\_set}_{i,f}$ (${Patent\_set}_{i,b}$) denotes the set of forward (backward) citations of ${patent}_{i}$, $\left\{category|\#\left({patent}_{m},ha{s}_{category},category\right)=1,{patent}_{m}\in {Patent\_set}_{i,f}\right\}$ is the set of unique categories to which these forward citations belong, and $\left|set\right|$ is the number of items in the $set$. ${PG}_{{patent}_{i}}$ (${PO}_{{patent}_{i}}$) counts the average number of unique categories to which these forward (backward) citations belong.

### Patent recommendation

In the patent recommendation module, we design an interpretable recommendation model based on DNN and LRP techniques. Given a set of patent-company pairs, the study transmits their connectivity and quality features to a basic DNN, a feed-forward neural network with multiple hidden layers. The DNN maps the features non-linearly from its input layer to a sequence of hidden layers and, at last, its output layer, which produces the probability that a specific company will select a specific patent. Let ${a}^{0}$ be the input vector of the input layer of an *L*-layer DNN and ${a}^{l}$ ($l=1, 2, 3, \dots , L$) be the output vector of its *l*-th layer (the output of the (*l*-1)-th layer is the input of the *l*-th layer). Then, the DNN is defined as follows:

10 $${a}^{l}={\phi }^{l}\left({\omega }^{l}{a}^{l-1}+{b}^{l}\right),$$

where ${\phi }^{l}\left(\right)$ represents an activation function that performs a nonlinear mapping from the (*l*-1)-th layer to the *l*-th layer, ${\omega }^{l}$ is a weight matrix for the mapping, and ${b}^{l}$ is a bias vector for the *l*-th layer. For hidden layers, we use the rectified linear unit (ReLU) as the activation function because it overcomes gradient vanish. The activation function of the output layer is Sigmoid because it constrains the output value within the range of (0, 1). After training the DNN, we use it to estimate the probabilities that target companies will select candidate patents and thus generate recommendation lists for the target companies.

The DNN is a black-box model, which fails to explain why certain patents are recommended to a target company. To empower the DNN with interpretability, we combine it with the LRP technique, which infers the relevance of input features to the output value of the DNN. The core mechanism of LRP is that it assigns relevance scores to the input features by tracing their contributions, layer by layer, back to the output value. Consequently, we can interpret recommendation results by describing which pieces of information dominate in generating each recommendation. The relevance of each input feature is inferred as follows:

11 $${r}_{i}^{l-1}={\sum }_{j}\left({a}_{i,j}^{l-1}/{\sum }_{k}{a}_{k,j}^{l-1}\right){r}_{j}^{l},$$

where ${r}_{j}^{l}$ is the relevance score of neuron $j$ of layer $l$, ${a}_{i,j}^{l-1}$ is the contribution made by neuron $i$ of layer $(l-1)$ to neuron $j$ of layer $l$ in the forward propagation, and ${\sum }_{k}{a}_{k,j}^{l-1}$ is the total contribution made by all neurons of layer $(l-1)$ to neuron $j$ of layer $l$ in the forward propagation. After obtaining the relevance scores of the input features for each recommendation, we can visualize them for better interpretation.


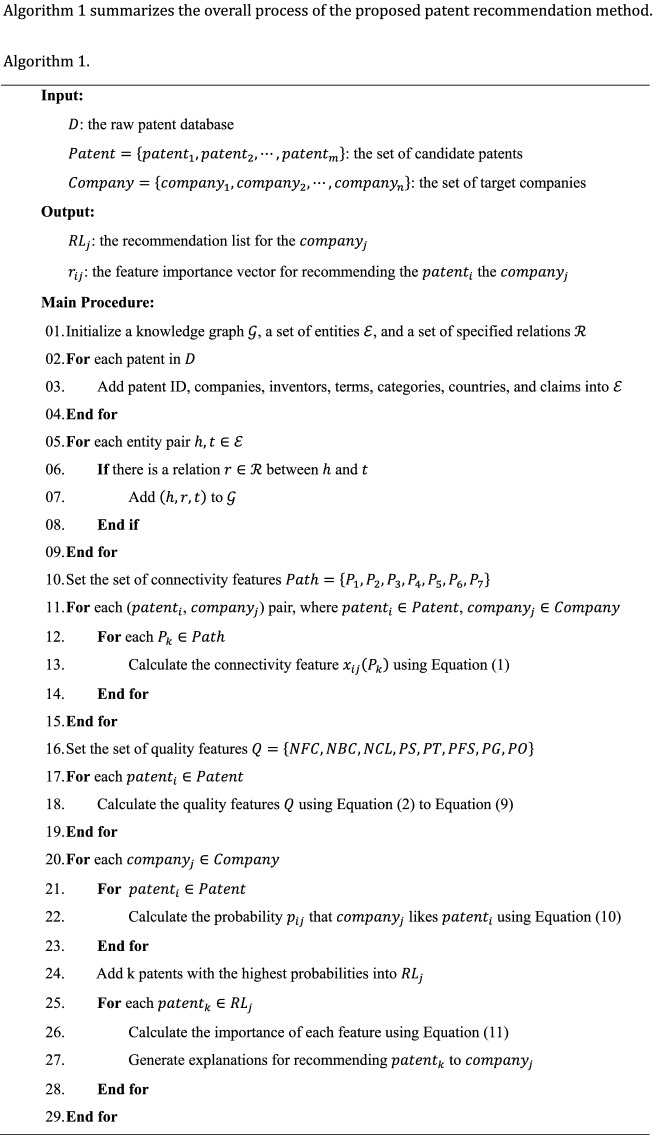


## Experimental evaluation

### Experimental setup

Experiments are conducted on a computer with a Windows 11 operating system and an AMD Ryzen 5 PRO 4650U @ 2.10 GHz CPU. We implement codes using Python, TensorFlow, and other tools or packages like NumPy, Keras, Pandas, etc. The model optimizer, learning rate, and batch size are set to the Adam optimizer, 0.001, and 128, respectively. Besides, we set the dropout rate of turning off neurons to 0.2 to avoid overfitting.

We evaluate the proposed patent recommendation method with real-world data from the USPTO and PatentsView databases. The former contains information of patent transactions, namely, which patents have been assigned to which patent assignees. The latter includes details of patents, such as their titles, abstracts, categories, inventors, and references. The two share the same identities of patents and thus can be merged based on the identities. To evaluate the proposed method, we randomly select 500 companies that have at least 50 records of patent assignments and collect all patents owned by the companies from the USPTO database. Then, we collect patent information from the PatentsView database, namely, inventors, claims, categories, references, terms, and protected countries. The collected information constitutes a knowledge graph for the selected companies and its statistics are reported in Table 3.

**Table 3:** Statistics of the knowledge graph.

Entity types	Number of instances	Relation types	Number of instances
Companies	227,074	is_transferred_to	1,819,490
Patents	907,661	cites	14,467,861
Countries	123	is_protected_in	1,123,087
Inventors	816,154	is_invented_by	2,229,005
Claims	14,548,355	has_claim	14,548,355
Categories	177,017	has_category	8,631,105
Terms	1,420,766	has_term	2,497,322

For each selected company, we consider the first 50% of patents it purchased as known information in the knowledge graph and keep the rest unknown for model training or testing. This study randomly chooses 70% of the selected companies, along with their hypothetically unknown patents, as training data and the rest as test data.

### Baseline methods

To evaluate the effectiveness of the proposed method (denoted as *Ours*), we compare it with the following baselines.

*DNN*+*C* a deep neural network with the connectivity features, which indicate the relevance between patents and companies. This method evaluates the role of the relevance in recommending suitable patents without considering their quality.

*DNN*+*Q* a deep neural network with the quality features, which reflect the quality of patents. The baseline evaluates the role of patent quality in recommending patents to companies without considering their relevance to the companies.

*CF* a collaborative filtering method that uses a user-item matrix to identify the nearest neighbors of a target user and leverages their preferences to infer the preference of the target user.

*CB* a content-based filtering method that represents patents and companies as vectors of terms and identifies relevant patents for target companies by calculating the cosine similarities of their vectors.

*SVM* a support vector machine model with the connectivity and quality features.

*RF* a random forest model with the connectivity and quality features.

*HIN* a patent recommendation method that leverages meta paths in knowledge graph for inferring the relevance between companies and patents^8^.

*RippleNet* a state-of-the-art recommendation method that leverages knowledge graph and deep learning for recommendations^45^.

Comparing the proposed method with the first two baselines reflects whether combing the connectivity and quality features outperforms using only one type of features. The remaining baselines are compared to evaluate whether the proposed method has superior performance in recommending patents to companies.

### Evaluation metrics

This study selects three common metrics to evaluate the recommendation performance, namely, precision, recall, and MAP. Given a target company, precision shows the percentage of recommended patents that were transferred to the company. Recall represents the ratio of correctly recommended patents to the patents that were transferred to the company. Precision and recall fail to consider the rankings of correctly recommended patents in recommendation lists. MAP overcomes the drawback by considering both the precision and the rankings of recommended patents. A high MAP means that many suitable patents are recommended and they rank high on recommendation lists. We use $RS$ to denote the set of patents recommended to the target company, $TS$ the set of patents that were transferred to the target company, and $RS\cap TS$ the set of patents in both $RS$ and $TS$. Then, we define the above metrics as follows:

12 $$precision@k=\frac{\left|RS\cap TS\right|}{\left|RS\right|},$$

13 $$recall@k=\frac{\left|RS\cap TS\right|}{\left|TS\right|},$$

14 $$MAP@k=\frac{1}{\left|RS\right|}\times \sum_{i=1}^{\left|RS\right|}\left[rel\left(i\right)\times precision@i\right],$$

where @*k* means the top *k* patents recommended to companies, $\left|RS\right|$ and $\left|TS\right|$ represent the number of patents in corresponding sets, and $rel\left(i\right)$ equals 1 if the $i$-th patent of $RS$ is in $TS$ and 0 otherwise.

## Results and discussions

### Recommendation results

To evaluate the effectiveness of the proposed method, we compare its recommendation performance with the baseline methods. We vary the number of recommended patents from 10 to 50 and present recommendation results in Table 4. The table uses bold numbers to represent the best performance of corresponding metrics and numbers in brackets to indicate the improvement of the proposed method compared to the best baseline method.

**Table 4:** Recommendation performance of different methods.

Metrics	Ours	CF	CB	SVM	RF	HIN	RippleNet
Precision@10	**0.841** (7.50%)	0.159	0.403	0.778	0.782	0.738	0.767
Precision@20	**0.667** (9.10%)	0.159	0.351	0.606	0.612	0.579	0.597
Precision@30	**0.556** (7.65%)	0.151	0.314	0.509	0.517	0.489	0.501
Precision@40	**0.484** (6.49%)	0.145	0.282	0.443	0.454	0.430	0.439
Precision@50	**0.430** (5.67%)	0.141	0.257	0.393	0.407	0.384	0.390
Recall@10	**0.468** (18.78%)	0.028	0.085	0.391	0.394	0.348	0.379
Recall@20	**0.608** (20.15%)	0.047	0.129	0.498	0.506	0.443	0.477
Recall@30	**0.667** (18.58%)	0.063	0.157	0.552	0.563	0.494	0.526
Recall@40	**0.706** (17.63%)	0.074	0.174	0.584	0.600	0.528	0.556
Recall@50	**0.730** (16.62%)	0.087	0.186	0.607	0.626	0.550	0.575
MAP@10	**0.829** (8.56%)	0.139	0.396	0.766	0.764	0.726	0.759
MAP@20	**0.655** (10.36%)	0.132	0.344	0.592	0.593	0.567	0.589
MAP@30	**0.545** (9.09%)	0.123	0.308	0.495	0.499	0.478	0.494
MAP@40	**0.473** (7.99%)	0.117	0.276	0.429	0.438	0.419	0.432
MAP@50	**0.419** (6.98%)	0.112	0.251	0.380	0.391	0.374	0.384

Bold numbers represent the best performance of corresponding metrics and numbers in brackets indicate the improvement of the proposed method compared to the best baseline method.

Several conclusions can be drawn from the above results. First, the proposed method outperforms the baseline methods in terms of precision, recall, and MAP. This demonstrates the effectiveness and the necessity of considering patent quality in recommending patents to companies. Second, the MAP improvements are higher than the improvements in precision. This means that considering patent quality not only helps to find more patents that are relevant to the companies but also helps to improve the rankings of relevant patents in the recommendation lists. Third, the CF method performs poorly for the patent recommendation. This result meets our expectations because most patents have been transferred to only one company. Hence, the CF method suffers from the data sparsity problem in this recommendation context. Fourth, CF and CB methods perform much worse than the other methods. One likely reason is that only a small part of patent information is involved in the two methods. Fifth, our method outperforms SVM and RF even though they use the same information. This indicates that the deep learning model can better capture companies’ preferences than the traditional machine learning models in the current context.

### Interpretability analysis

**Figure 3:**
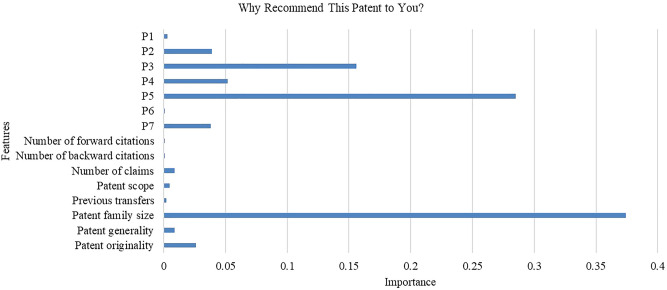
An illustration of the visualization module.

We conduct qualitative analysis to evaluate the interpretability of our method. Specifically, we design a module that visualizes the importance of features to each recommendation. Figure 3 illustrates the visualization of a randomly selected company and a patent recommended to it. The figure shows the main information based on which the patent is recommended to the company. Specifically, patent family size is the overriding reason, followed by *P*_*5*_ and *P*_*3*_, which can be referred to Table 2. The results show that being protected in multiple countries, having a direct citation relation, as well as having common inventors, with the patents owned by the company are the main reasons why the patent is recommended. In contrast, *P*_*6*_, the number of forward citations, and the number of backward citations barely impact the recommendation. Averagely, the connectivity features contribute more to the recommendation than the quality features.

### Ablation study

**Figure 4:**
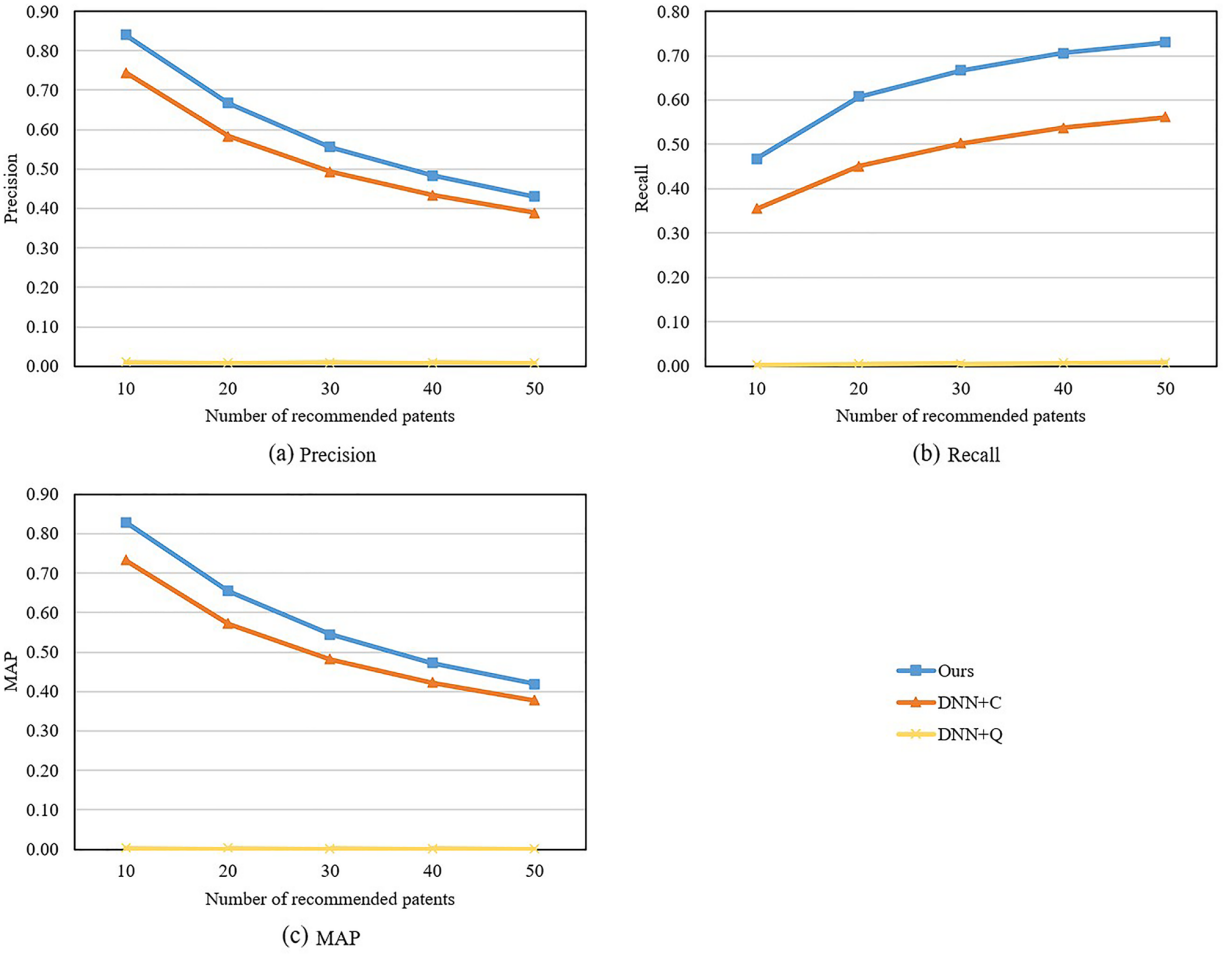
Recommendation performance of the proposed method with different features.

To analyze the impacts of key components of the proposed method in generating accurate and interpretable results, we conduct additional experiments. First, to examine the role of connectivity and quality features in patent recommendation, we experiment with the proposed method using different features and present results in Fig. 4. The results show that connectivity features generate good performance, meaning that they are helpful in finding relevant patents for companies. On the contrary, the recommendation performance of DNN+Q is about 0, meaning that quality features alone lead to very bad performance. This is because many high-quality patents are irrelevant to the business of target companies and are unnecessary for the companies. Consequently, recommending these patents to the target companies results in low performance. Besides, combining both types of features yields the best performance. This confirms the need of considering both types of features in patent recommendation.

**Figure 5:**
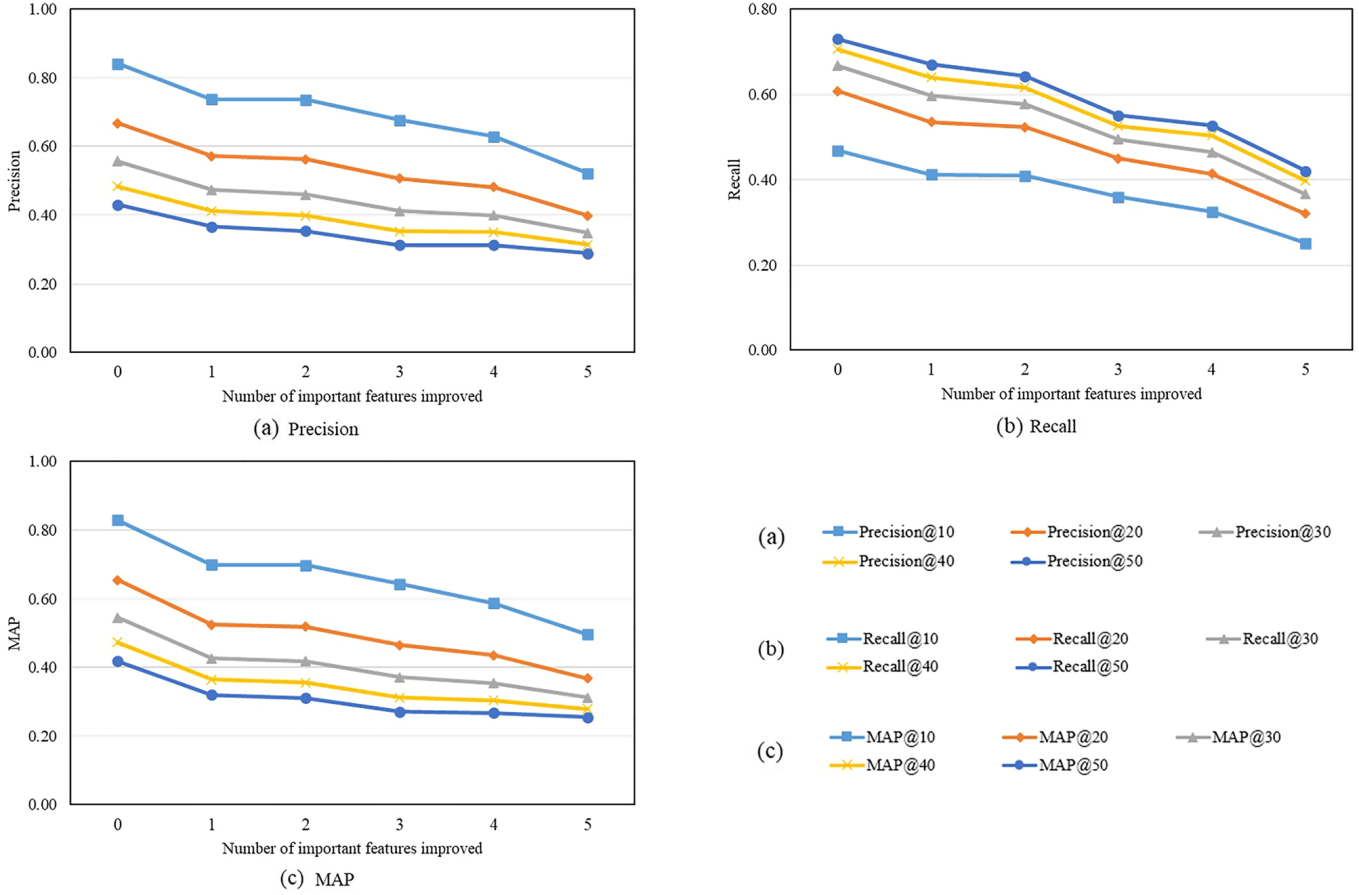
Recommendation performance after removing important features.

Second, we examine the impacts of important features on recommendation performance. This also helps to evaluate the interpretability of our method. To this end, we remove features identified as important from the proposed method and track its changes in recommendation performance. Specifically, we remove the top five most important features one by one and measure the resulting recommendation performance, which is presented in Fig. 5. Results show that the recommendation performance decreases as the number of removed features increases. However, the drops in performance may simply be caused by discarding features. To demonstrate whether removing the identified important features leads to larger drops compared to removing the unimportant features, we remove the least important features one by one and calculate the resulting recommendation performance.

**Figure 6:**
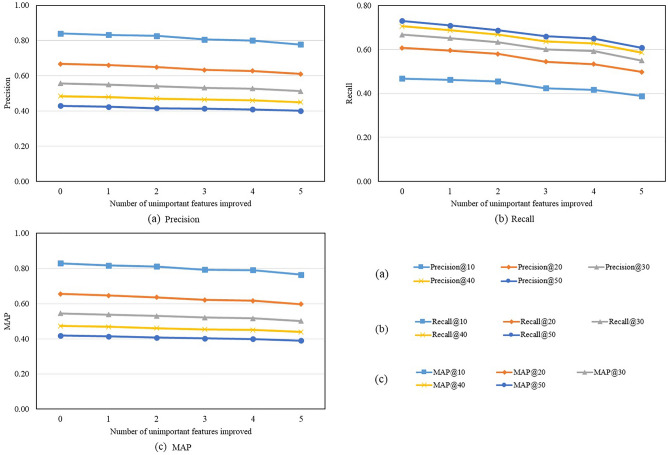
Recommendation performance after removing unimportant features.

Figure 6 presents the recommendation results of removing the least important features one by one. The results show that removing the least important features also leads to drops in recommendation performance, but the drops are much smaller than those of removing the most important features. Besides, the performance difference between removing important and unimportant features widens as the number of removed features increases. These observations show that the identified important features do contribute to the recommendations and removing them leads to dramatic drops in precision, recall, and MAP. The results confirm that the identified important features are more significant to recommendation results and can be used to interpret the results.

Based on the above analysis and discussion, we conclude the impact of the proposed method on the research problems. Regarding the problem that previous patent recommendation methods ignore patent quality, the proposed method applies eight quality features to quantify patent quality and involves it in the recommendation process. Experimental results demonstrate that considering patent quality improves patent recommendation performance. In terms of lacking interpretability, the proposed method addresses it by combining a deep neural network with a relevance propagation technique, which can identify important features to each recommendation. Consequently, our method can generate explanations based on the important features and thus ease the lacking of interpretability.

## Conclusions

In this study, we propose an interpretable patent recommendation method based on knowledge graph and deep learning. The proposed method organizes various patent information as a knowledge graph. It then defines and extracts connectivity and quality features that indicate the relevance of patent-company pairs and patent quality. Given the extracted features, we design an interpretable recommendation model by combining DNN and LRP techniques. We conduct experiments to evaluate the proposed method and experimental results show that its average precision@k, recall@k, and MAP@k (k = 10, 20, 30, 40, 50) are 0.596, 0.636, and 0.584, respectively. The results are 7.28%, 18.35%, and 8.60% higher than those of best baseline methods. We qualitatively and quantitatively analyze the interpretability of the proposed method. The qualitative and quantitative analyses show that our method can provide interpretable results.

This research extends existing studies on patent recommendations. First, this is the first study that combines knowledge graph, DNN, and LRP techniques for effective and interpretable patent recommendations. Knowledge graph models heterogeneous patent data and facilitates feature extraction and interpretation. DNN enables better patent recommendation by capturing nonlinear and nontrivial relationships between patents and companies. LRP endows our patent recommendation model with interpretability by identifying features that contribute the most to recommendation results. Experiments show that our method is superior to baseline methods. Second, this research incorporates patent quality into patent recommendation. Experiments demonstrate its effectiveness in enhancing recommendation performance. Third, this study provides a practical solution to facilitate technology transfer. Through the proposed patent recommendation method, companies can find suitable patents with explanations.

Future research can extend this study from different aspects. The first one is to incorporate more company information into the patent recommendation model. For example, the industries and business scopes of companies can be used to identify relevant patents. Future work can include such information into knowledge graph and extract more features for patent recommendations. The second one is to include patent portfolios into patent recommendations because companies may consider their existing patents when obtaining new ones. Future research can design new recommendation models with features that indicate the relationships between candidate patents and the existing patents.

## Data Availability

The data used in this paper are public and can be downloaded from the following links: https://www.uspto.gov/learning-and-resources/electronic-data-products/patent-assignment-dataset and http://www.patentsview.org/download/.
